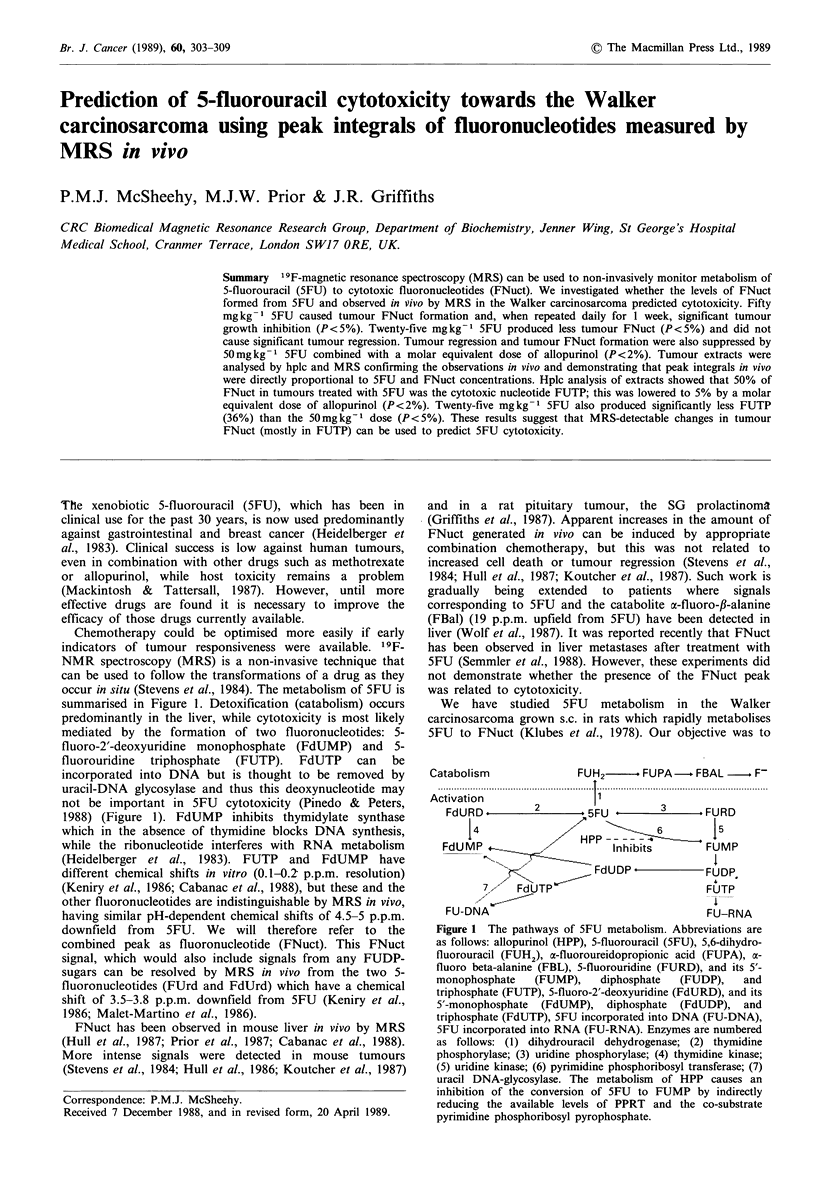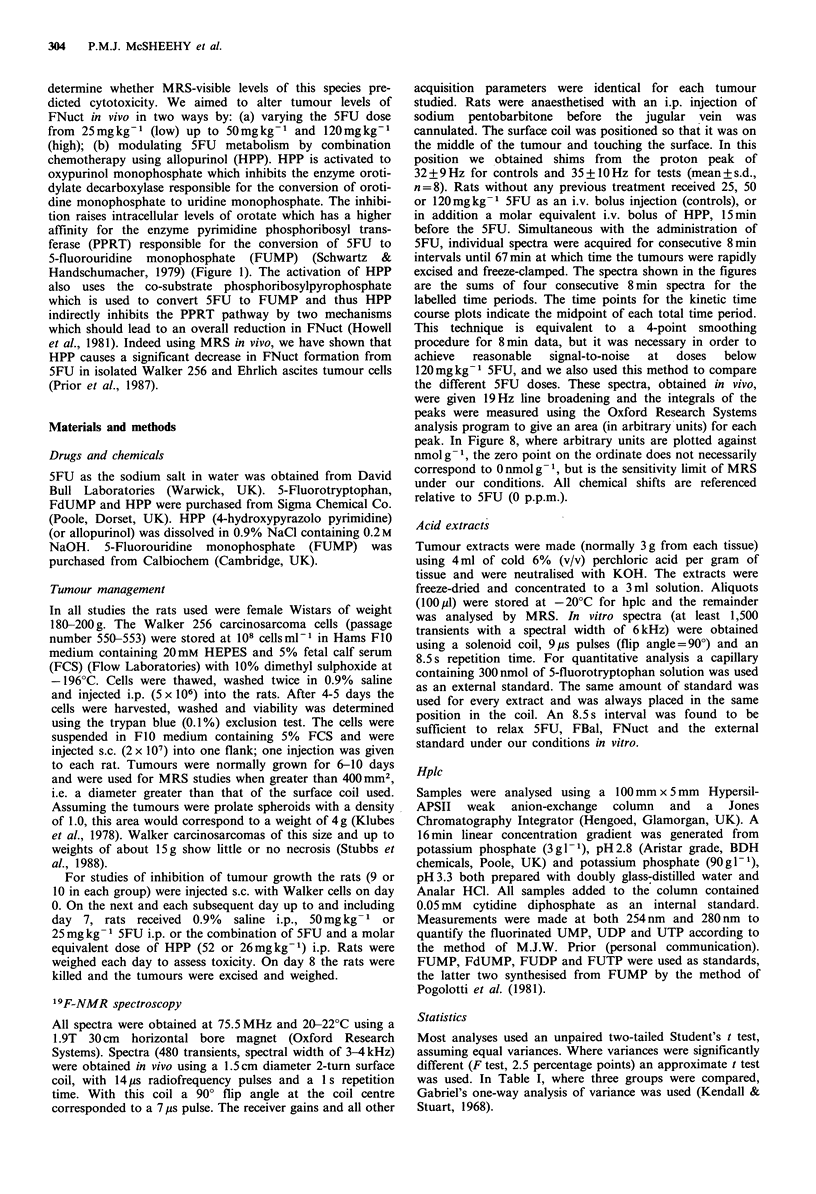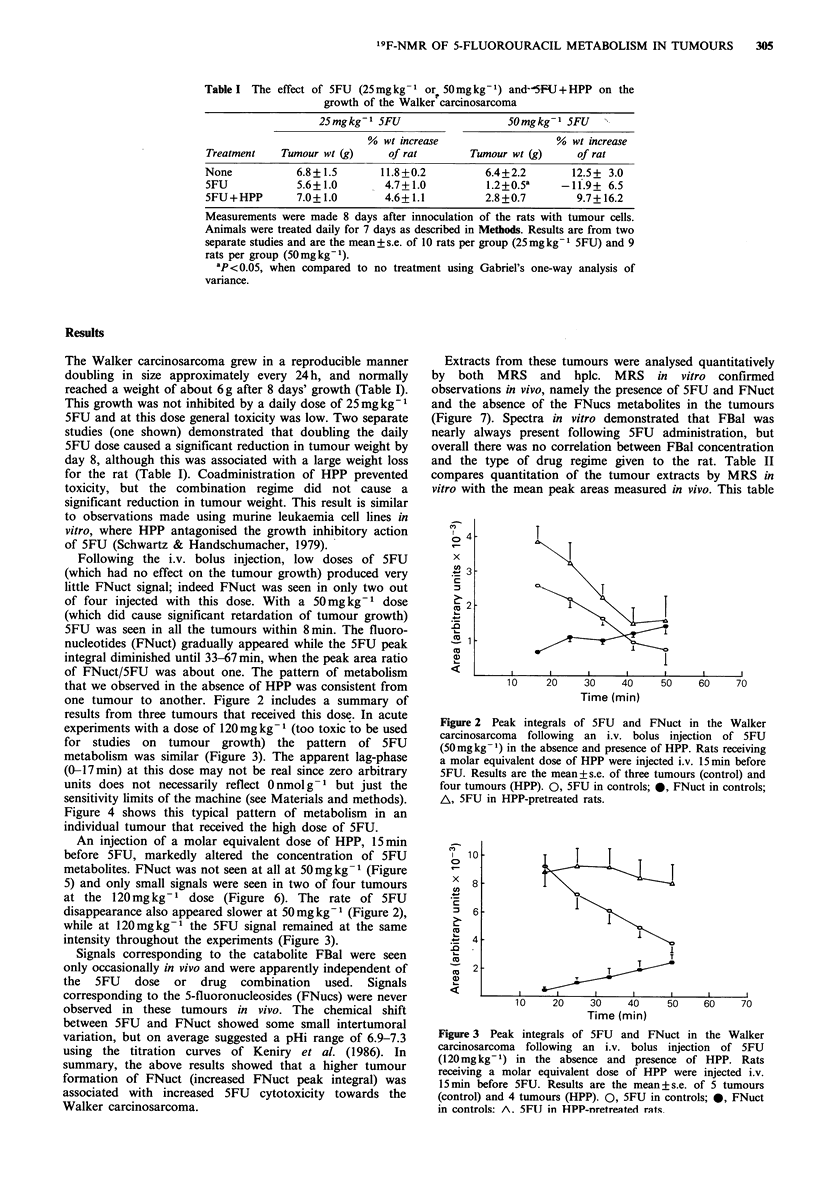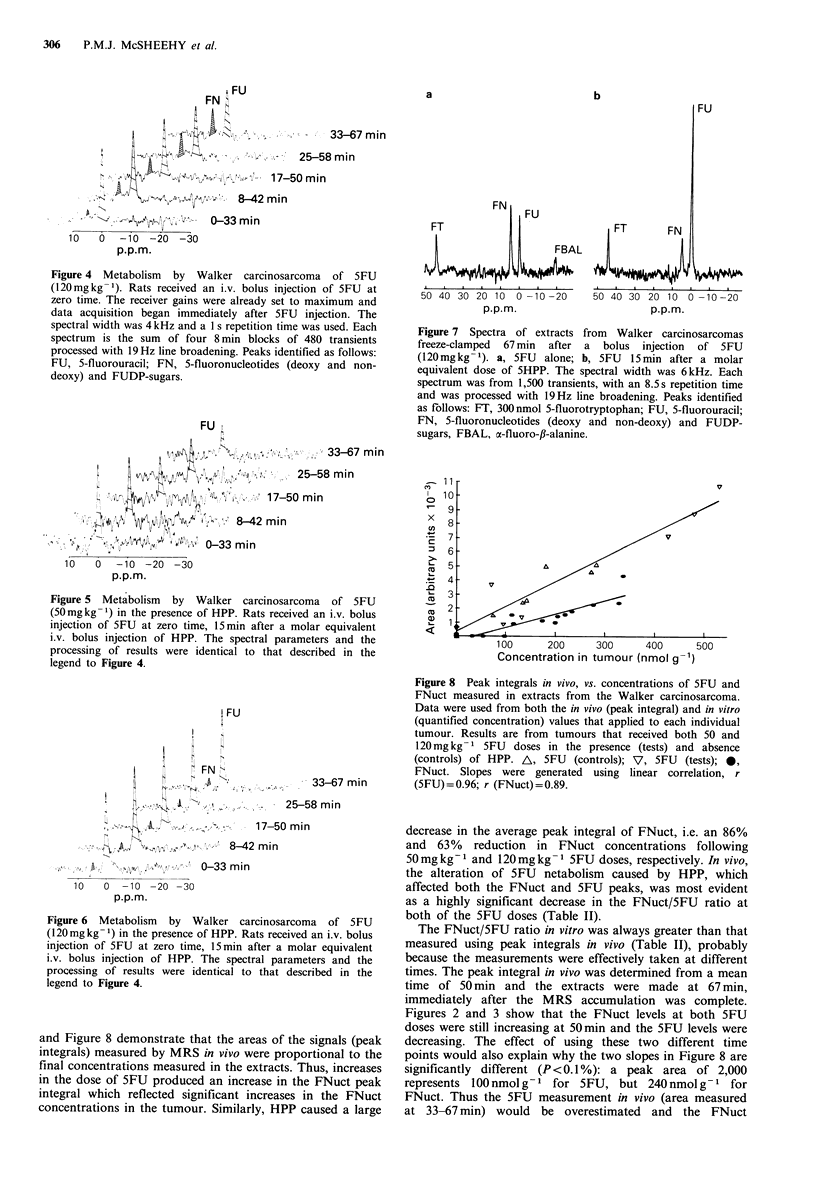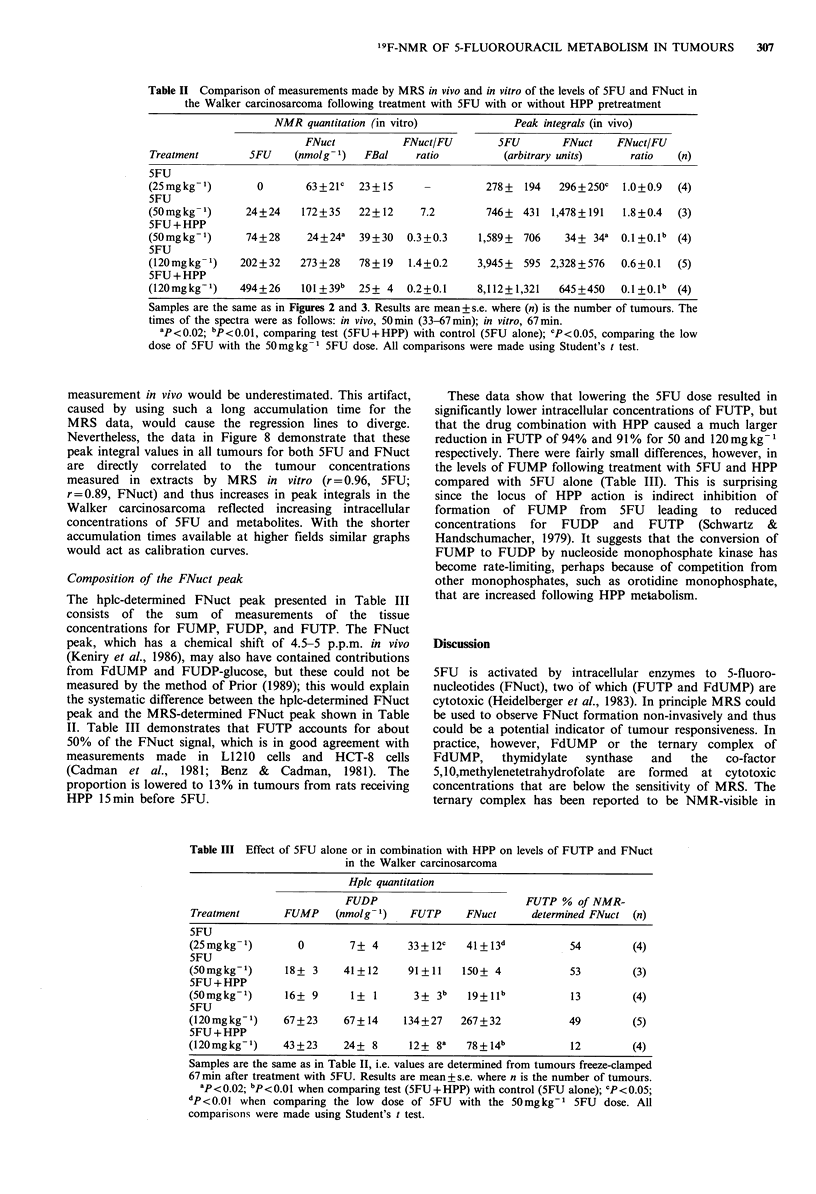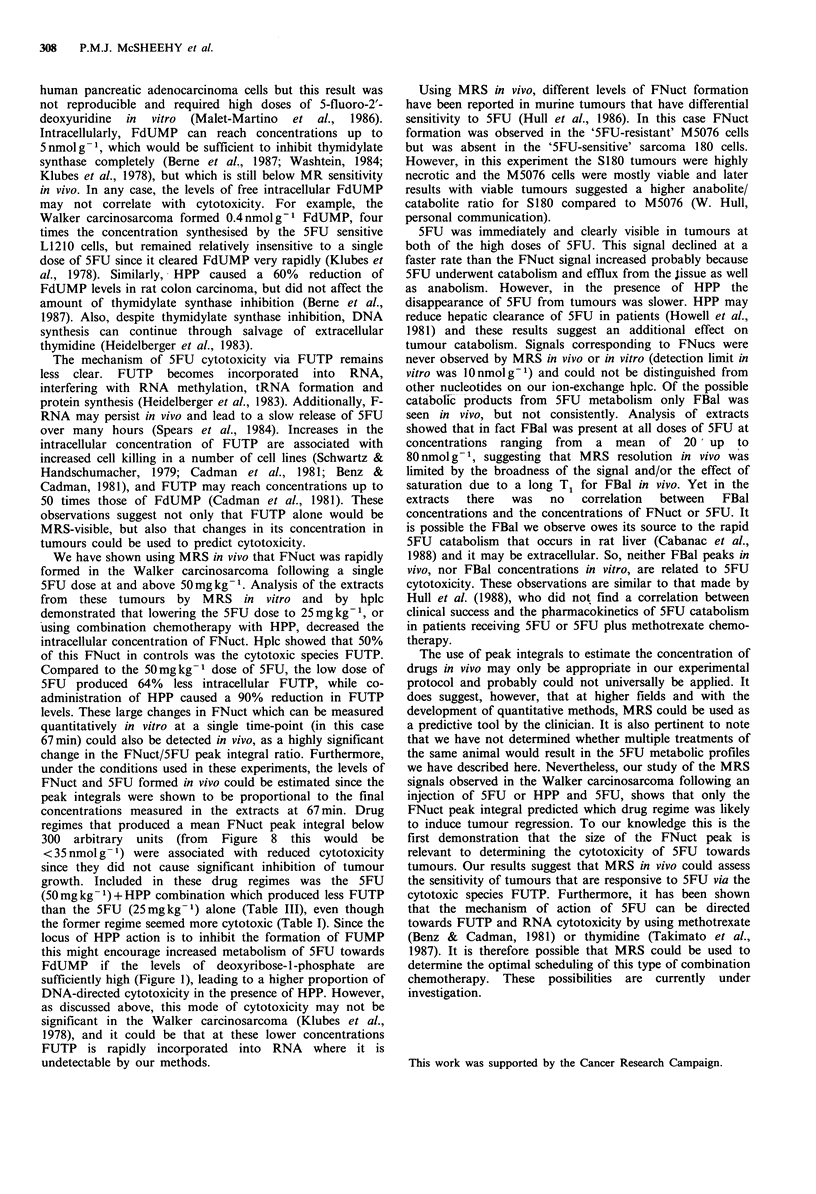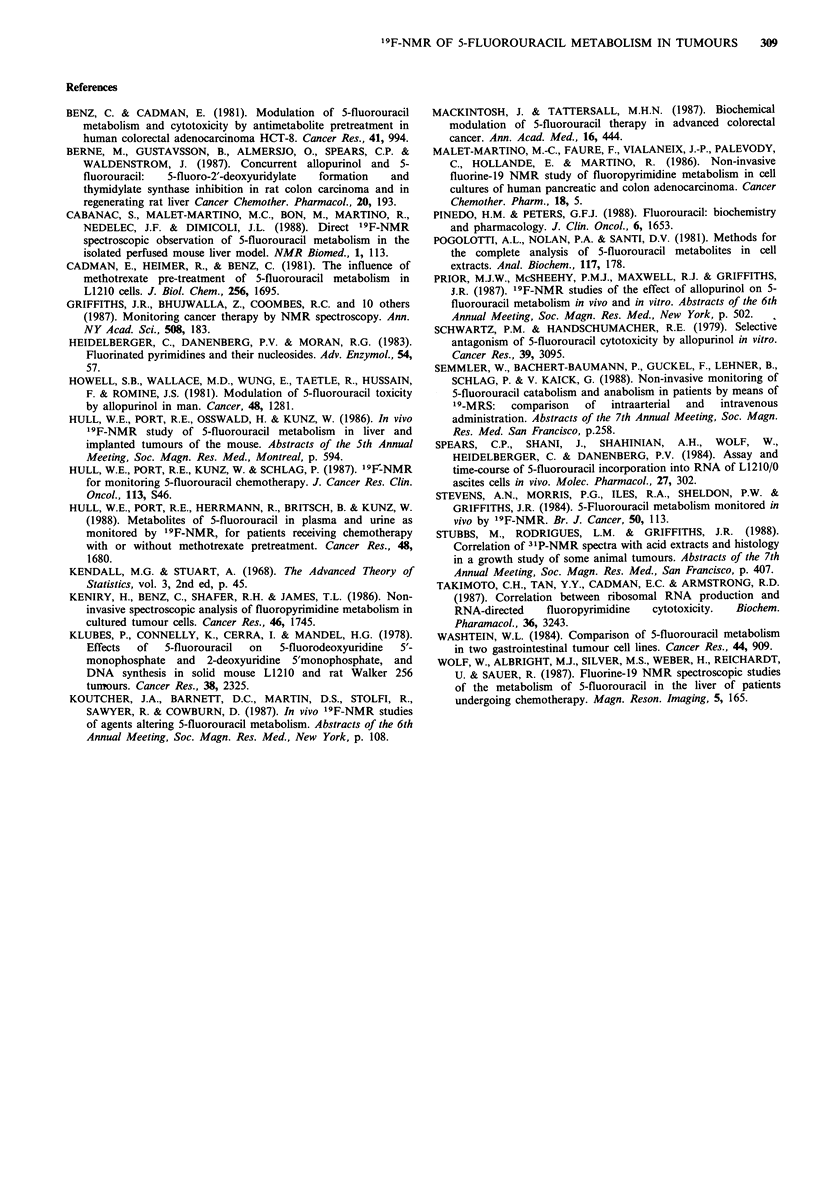# Prediction of 5-fluorouracil cytotoxicity towards the Walker carcinosarcoma using peak integrals of fluoronucleotides measured by MRS in vivo.

**DOI:** 10.1038/bjc.1989.275

**Published:** 1989-09

**Authors:** P. M. McSheehy, M. J. Prior, J. R. Griffiths

**Affiliations:** Department of Biochemistry, St George's Hospital Medical School, London, UK.

## Abstract

19F-magnetic resonance spectroscopy (MRS) can be used to non-invasively monitor metabolism of 5-fluorouracil (5FU) to cytotoxic fluoronucleotides (FNuct). We investigated whether the levels of FNuct formed from 5FU and observed in vivo by MRS in the Walker carcinosarcoma predicted cytotoxicity. Fifty mg kg-1 5FU caused tumour FNuct formation and, when repeated daily for 1 week, significant tumour growth inhibition (P less than 5%). Twenty-five mg kg-1 5FU produced less tumour FNuct (P less than 5%) and did not cause significant tumour regression. Tumour regression and tumour FNuct formation were also suppressed by 50 mg kg-1 5FU combined with a molar equivalent dose of allopurinol (P less than 2%). Tumour extracts were analysed by hplc and MRS confirming the observations in vivo and demonstrating that peak integrals in vivo were directly proportional to 5FU and FNuct concentrations. Hplc analysis of extracts showed that 50% of FNuct in tumours treated with 5FU was the cytotoxic nucleotide FUTP; this was lowered to 5% by a molar equivalent dose of allopurinol (P less than 2%). Twenty-five mg kg-1 5FU also produced significantly less FUTP (36%) than the 50 mg kg-1 dose (P less than 5%). These results suggest that MRS-detectable changes in tumour FNuct (mostly in FUTP) can be used to predict 5FU cytotoxicity.


Br. J. Cancer (1989), 60, 303–309 © The Macmillan Press Ltd., 1989

### Prediction of 5-fluorouracil cytotoxicity towards the Walker carcinosarcoma using peak integrals of fluoronucleotides measured by MRS *in vivo*

P.M.J. McSheehy, M.J.W. Prior & J.R. Griffiths

*CRC Biomedical Magnetic Resonance Research Group, Department of Biochemistry, Jenner Wing, St George's Hospital Medical School, Cranmer Terrace, London SW17 0RE, UK.*

**Summary** $^{19}$F-magnetic resonance spectroscopy (MRS) can be used to non-invasively monitor metabolism of 5-fluorouracil (5FU) to cytotoxic fluoronucleotides (FNuct). We investigated whether the levels of FNuct formed from 5FU and observed *in vivo* by MRS in the Walker carcinosarcoma predicted cytotoxicity. Fifty $mg\,kg^{-1}$ 5FU caused tumour FNuct formation and, when repeated daily for 1 week, significant tumour growth inhibition ($P<5\%$). Twenty-five $mg\,kg^{-1}$ 5FU produced less tumour FNuct ($P<5\%$) and did not cause significant tumour regression. Tumour regression and tumour FNuct formation were also suppressed by $50\,mg\,kg^{-1}$ 5FU combined with a molar equivalent dose of allopurinol ($P<2\%$). Tumour extracts were analysed by hplc and MRS confirming the observations *in vivo* and demonstrating that peak integrals *in vivo* were directly proportional to 5FU and FNuct concentrations. Hplc analysis of extracts showed that 50% of FNuct in tumours treated with 5FU was the cytotoxic nucleotide FUTP; this was lowered to 5% by a molar equivalent dose of allopurinol ($P<2\%$). Twenty-five $mg\,kg^{-1}$ 5FU also produced significantly less FUTP (36%) than the $50\,mg\,kg^{-1}$ dose ($P<5\%$). These results suggest that MRS-detectable changes in tumour FNuct (mostly in FUTP) can be used to predict 5FU cytotoxicity.

The xenobiotic 5-fluorouracil (5FU), which has been in clinical use for the past 30 years, is now used predominantly against gastrointestinal and breast cancer (Heidelberger *et al.*, 1983). Clinical success is low against human tumours, even in combination with other drugs such as methotrexate or allopurinol, while host toxicity remains a problem (Mackintosh & Tattersall, 1987). However, until more effective drugs are found it is necessary to improve the efficacy of those drugs currently available.

Chemotherapy could be optimised more easily if early indicators of tumour responsiveness were available. $^{19}$F-NMR spectroscopy (MRS) is a non-invasive technique that can be used to follow the transformations of a drug as they occur *in situ* (Stevens *et al.*, 1984). The metabolism of 5FU is summarised in Figure 1. Detoxification (catabolism) occurs predominantly in the liver, while cytotoxicity is most likely mediated by the formation of two fluoronucleotides: 5-fluoro-2′-deoxyuridine monophosphate (FdUMP) and 5-fluorouridine triphosphate (FUTP). FdUTP can be incorporated into DNA but is thought to be removed by uracil-DNA glycosylase and thus this deoxynucleotide may not be important in 5FU cytotoxicity (Pinedo & Peters, 1988) (Figure 1). FdUMP inhibits thymidylate synthase which in the absence of thymidine blocks DNA synthesis, while the ribonucleotide interferes with RNA metabolism (Heidelberger *et al.*, 1983). FUTP and FdUMP have different chemical shifts *in vitro* (0.1–0.2 p.p.m. resolution) (Keniry *et al.*, 1986; Cabanac *et al.*, 1988), but these and the other fluoronucleotides are indistinguishable by MRS *in vivo*, having similar pH-dependent chemical shifts of 4.5–5 p.p.m. downfield from 5FU. We will therefore refer to the combined peak as fluoronucleotide (FNuct). This FNuct signal, which would also include signals from any FUDP-sugars can be resolved by MRS *in vivo* from the two 5-fluoronucleotides (FUrd and FdUrd) which have a chemical shift of 3.5–3.8 p.p.m. downfield from 5FU (Keniry *et al.*, 1986; Malet-Martino *et al.*, 1986).

FNuct has been observed in mouse liver *in vivo* by MRS (Hull *et al.*, 1987; Prior *et al.*, 1987; Cabanac *et al.*, 1988). More intense signals were detected in mouse tumours (Stevens *et al.*, 1984; Hull *et al.*, 1986; Koutcher *et al.*, 1987) and in a rat pituitary tumour, the SG prolactinoma (Griffiths *et al.*, 1987). Apparent increases in the amount of FNuct generated *in vivo* can be induced by appropriate combination chemotherapy, but this was not related to increased cell death or tumour regression (Stevens *et al.*, 1984; Hull *et al.*, 1987; Koutcher *et al.*, 1987). Such work is gradually being extended to patients where signals corresponding to 5FU and the catabolite α-fluoro-β-alanine (FBal) (19 p.p.m. upfield from 5FU) have been detected in liver (Wolf *et al.*, 1987). It was reported recently that FNuct has been observed in liver metastases after treatment with 5FU (Semmler *et al.*, 1988). However, these experiments did not demonstrate whether the presence of the FNuct peak was related to cytotoxicity.

We have studied 5FU metabolism in the Walker carcinosarcoma grown s.c. in rats which rapidly metabolises 5FU to FNuct (Klubes *et al.*, 1978). Our objective was to

**Figure 1** The pathways of 5FU metabolism. Abbreviations are as follows: allopurinol (HPP), 5-fluorouracil (5FU), 5,6-dihydrofluorouracil ($FUH_2$), α-fluoroureidopropionic acid (FUPA), α-fluoro beta-alanine (FBL), 5-fluorouridine (FURD), and its 5′-monophosphate (FUMP), diphosphate (FUDP), and triphosphate (FUTP), 5-fluoro-2′-deoxyuridine (FdURD), and its 5′-monophosphate (FdUMP), diphosphate (FdUDP), and triphosphate (FdUTP), 5FU incorporated into DNA (FU-DNA), 5FU incorporated into RNA (FU-RNA). Enzymes are numbered as follows: (1) dihydrouracil dehydrogenase; (2) thymidine phosphorylase; (3) uridine phosphorylase; (4) thymidine kinase; (5) uridine kinase; (6) pyrimidine phosphoribosyl transferase; (7) uracil DNA-glycosylase. The metabolism of HPP causes an inhibition of the conversion of 5FU to FUMP by indirectly reducing the available levels of PPRT and the co-substrate pyrimidine phosphoribosyl pyrophosphate.

Correspondence: P.M.J. McSheehy.
Received 7 December 1988, and in revised form, 20 April 1989.

determine whether MRS-visible levels of this species predicted cytotoxicity. We aimed to alter tumour levels of FNuct *in vivo* in two ways by: (a) varying the 5FU dose from 25 mg kg$^{-1}$ (low) up to 50 mg kg$^{-1}$ and 120 mg kg$^{-1}$ (high); (b) modulating 5FU metabolism by combination chemotherapy using allopurinol (HPP). HPP is activated to oxypurinol monophosphate which inhibits the enzyme orotidylate decarboxylase responsible for the conversion of orotidine monophosphate to uridine monophosphate. The inhibition raises intracellular levels of orotate which has a higher affinity for the enzyme pyrimidine phosphoribosyl transferase (PPRT) responsible for the conversion of 5FU to 5-fluorouridine monophosphate (FUMP) (Schwartz & Handschumacher, 1979) (Figure 1). The activation of HPP also uses the co-substrate phosphoribosylpyrophosphate which is used to convert 5FU to FUMP and thus HPP indirectly inhibits the PPRT pathway by two mechanisms which should lead to an overall reduction in FNuct (Howell *et al.*, 1981). Indeed using MRS *in vivo*, we have shown that HPP causes a significant decrease in FNuct formation from 5FU in isolated Walker 256 and Ehrlich ascites tumour cells (Prior *et al.*, 1987).

### Materials and methods

### *Drugs and chemicals*

5FU as the sodium salt in water was obtained from David Bull Laboratories (Warwick, UK). 5-Fluorotryptophan, FdUMP and HPP were purchased from Sigma Chemical Co. (Poole, Dorset, UK). HPP (4-hydroxypyrazolo pyrimidine) (or allopurinol) was dissolved in 0.9% NaCl containing 0.2 M NaOH. 5-Fluorouridine monophosphate (FUMP) was purchased from Calbiochem (Cambridge, UK).

### *Tumour management*

In all studies the rats used were female Wistars of weight 180–200 g. The Walker 256 carcinosarcoma cells (passage number 550–553) were stored at $10^8$ cells ml$^{-1}$ in Hams F10 medium containing 20 mM HEPES and 5% fetal calf serum (FCS) (Flow Laboratories) with 10% dimethyl sulphoxide at −196°C. Cells were thawed, washed twice in 0.9% saline and injected i.p. ($5 \times 10^6$) into the rats. After 4-5 days the cells were harvested, washed and viability was determined using the trypan blue (0.1%) exclusion test. The cells were suspended in F10 medium containing 5% FCS and were injected s.c. ($2 \times 10^7$) into one flank; one injection was given to each rat. Tumours were normally grown for 6–10 days and were used for MRS studies when greater than 400 mm$^2$, i.e. a diameter greater than that of the surface coil used. Assuming the tumours were prolate spheroids with a density of 1.0, this area would correspond to a weight of 4 g (Klubes *et al.*, 1978). Walker carcinosarcomas of this size and up to weights of about 15 g show little or no necrosis (Stubbs *et al.*, 1988).

For studies of inhibition of tumour growth the rats (9 or 10 in each group) were injected s.c. with Walker cells on day 0. On the next and each subsequent day up to and including day 7, rats received 0.9% saline i.p., 50 mg kg$^{-1}$ or 25 mg kg$^{-1}$ 5FU i.p. or the combination of 5FU and a molar equivalent dose of HPP (52 or 26 mg kg$^{-1}$) i.p. Rats were weighed each day to assess toxicity. On day 8 the rats were killed and the tumours were excised and weighed.

### *$^{19}$F-NMR spectroscopy*

All spectra were obtained at 75.5 MHz and 20–22°C using a 1.9T 30 cm horizontal bore magnet (Oxford Research Systems). Spectra (480 transients, spectral width of 3–4 kHz) were obtained *in vivo* using a 1.5 cm diameter 2-turn surface coil, with 14 $\mu$s radiofrequency pulses and a 1 s repetition time. With this coil a 90° flip angle at the coil centre corresponded to a 7 $\mu$s pulse. The receiver gains and all other acquisition parameters were identical for each tumour studied. Rats were anaesthetised with an i.p. injection of sodium pentobarbitone before the jugular vein was cannulated. The surface coil was positioned so that it was on the middle of the tumour and touching the surface. In this position we obtained shims from the proton peak of $32 \pm 9$ Hz for controls and $35 \pm 10$ Hz for tests (mean ± s.d., $n = 8$). Rats without any previous treatment received 25, 50 or 120 mg kg$^{-1}$ 5FU as an i.v. bolus injection (controls), or in addition a molar equivalent i.v. bolus of HPP, 15 min before the 5FU. Simultaneous with the administration of 5FU, individual spectra were acquired for consecutive 8 min intervals until 67 min at which time the tumours were rapidly excised and freeze-clamped. The spectra shown in the figures are the sums of four consecutive 8 min spectra for the labelled time periods. The time points for the kinetic time course plots indicate the midpoint of each total time period. This technique is equivalent to a 4-point smoothing procedure for 8 min data, but it was necessary in order to achieve reasonable signal-to-noise at doses below 120 mg kg$^{-1}$ 5FU, and we also used this method to compare the different 5FU doses. These spectra, obtained *in vivo*, were given 19 Hz line broadening and the integrals of the peaks were measured using the Oxford Research Systems analysis program to give an area (in arbitrary units) for each peak. In Figure 8, where arbitrary units are plotted against nmol g$^{-1}$, the zero point on the ordinate does not necessarily correspond to 0 nmol g$^{-1}$, but is the sensitivity limit of MRS under our conditions. All chemical shifts are referenced relative to 5FU (0 p.p.m.).

### *Acid extracts*

Tumour extracts were made (normally 3 g from each tissue) using 4 ml of cold 6% (v/v) perchloric acid per gram of tissue and were neutralised with KOH. The extracts were freeze-dried and concentrated to a 3 ml solution. Aliquots (100 $\mu$l) were stored at −20°C for hplc and the remainder was analysed by MRS. *In vitro* spectra (at least 1,500 transients with a spectral width of 6 kHz) were obtained using a solenoid coil, 9 $\mu$s pulses (flip angle = 90°) and an 8.5 s repetition time. For quantitative analysis a capillary containing 300 nmol of 5-fluorotryptophan solution was used as an external standard. The same amount of standard was used for every extract and was always placed in the same position in the coil. An 8.5 s interval was found to be sufficient to relax 5FU, FBal, FNuct and the external standard under our conditions *in vitro*.

### *Hplc*

Samples were analysed using a 100 mm × 5 mm Hypersil-APSII weak anion-exchange column and a Jones Chromatography Integrator (Hengoed, Glamorgan, UK). A 16 min linear concentration gradient was generated from potassium phosphate (3 g l$^{-1}$), pH 2.8 (Aristar grade, BDH chemicals, Poole, UK) and potassium phosphate (90 g l$^{-1}$), pH 3.3 both prepared with doubly glass-distilled water and Analar HCl. All samples added to the column contained 0.05 mM cytidine diphosphate as an internal standard. Measurements were made at both 254 nm and 280 nm to quantify the fluorinated UMP, UDP and UTP according to the method of M.J.W. Prior (personal communication). FUMP, FdUMP, FUDP and FUTP were used as standards, the latter two synthesised from FUMP by the method of Pogolotti *et al.* (1981).

### *Statistics*

Most analyses used an unpaired two-tailed Student's *t* test, assuming equal variances. Where variances were significantly different (*F* test, 2.5 percentage points) an approximate *t* test was used. In Table I, where three groups were compared, Gabriel's one-way analysis of variance was used (Kendall & Stuart, 1968).

**Table I** The effect of 5FU (25 mg kg$^{-1}$ or 50 mg kg$^{-1}$) and 5FU+HPP on the growth of the Walker carcinosarcoma

| | *25 mg kg$^{-1}$ 5FU* | | *50 mg kg$^{-1}$ 5FU* | |
|---|---|---|---|---|
| *Treatment* | *Tumour wt (g)* | *% wt increase of rat* | *Tumour wt (g)* | *% wt increase of rat* |
| None | 6.8±1.5 | 11.8±0.2 | 6.4±2.2 | 12.5± 3.0 |
| 5FU | 5.6±1.0 | 4.7±1.0 | 1.2±0.5[a] | −11.9± 6.5 |
| 5FU+HPP | 7.0±1.0 | 4.6±1.1 | 2.8±0.7 | 9.7±16.2 |

Measurements were made 8 days after innoculation of the rats with tumour cells. Animals were treated daily for 7 days as described in **Methods**. Results are from two separate studies and are the mean±s.e. of 10 rats per group (25 mg kg$^{-1}$ 5FU) and 9 rats per group (50 mg kg$^{-1}$).

[a]$P<0.05$, when compared to no treatment using Gabriel's one-way analysis of variance.

### Results

The Walker carcinosarcoma grew in a reproducible manner doubling in size approximately every 24 h, and normally reached a weight of about 6 g after 8 days' growth (Table I). This growth was not inhibited by a daily dose of 25 mg kg$^{-1}$ 5FU and at this dose general toxicity was low. Two separate studies (one shown) demonstrated that doubling the daily 5FU dose caused a significant reduction in tumour weight by day 8, although this was associated with a large weight loss for the rat (Table I). Coadministration of HPP prevented toxicity, but the combination regime did not cause a significant reduction in tumour weight. This result is similar to observations made using murine leukaemia cell lines *in vitro*, where HPP antagonised the growth inhibitory action of 5FU (Schwartz & Handschumacher, 1979).

Following the i.v. bolus injection, low doses of 5FU (which had no effect on the tumour growth) produced very little FNuct signal; indeed FNuct was seen in only two out of four injected with this dose. With a 50 mg kg$^{-1}$ dose (which did cause significant retardation of tumour growth) 5FU was seen in all the tumours within 8 min. The fluoronucleotides (FNuct) gradually appeared while the 5FU peak integral diminished until 33–67 min, when the peak area ratio of FNuct/5FU was about one. The pattern of metabolism that we observed in the absence of HPP was consistent from one tumour to another. Figure 2 includes a summary of results from three tumours that received this dose. In acute experiments with a dose of 120 mg kg$^{-1}$ (too toxic to be used for studies on tumour growth) the pattern of 5FU metabolism was similar (Figure 3). The apparent lag-phase (0–17 min) at this dose may not be real since zero arbitrary units does not necessarily reflect 0 nmol g$^{-1}$ but just the sensitivity limits of the machine (see Materials and methods). Figure 4 shows this typical pattern of metabolism in an individual tumour that received the high dose of 5FU.

An injection of a molar equivalent dose of HPP, 15 min before 5FU, markedly altered the concentration of 5FU metabolites. FNuct was not seen at all at 50 mg kg$^{-1}$ (Figure 5) and only small signals were seen in two of four tumours at the 120 mg kg$^{-1}$ dose (Figure 6). The rate of 5FU disappearance also appeared slower at 50 mg kg$^{-1}$ (Figure 2), while at 120 mg kg$^{-1}$ the 5FU signal remained at the same intensity throughout the experiments (Figure 3).

Signals corresponding to the catabolite FBal were seen only occasionally *in vivo* and were apparently independent of the 5FU dose or drug combination used. Signals corresponding to the 5-fluoronucleosides (FNucs) were never observed in these tumours *in vivo*. The chemical shift between 5FU and FNuct showed some small intertumoral variation, but on average suggested a pHi range of 6.9–7.3 using the titration curves of Keniry *et al.* (1986). In summary, the above results showed that a higher tumour formation of FNuct (increased FNuct peak integral) was associated with increased 5FU cytotoxicity towards the Walker carcinosarcoma.

Extracts from these tumours were analysed quantitatively by both MRS and hplc. MRS *in vitro* confirmed observations *in vivo*, namely the presence of 5FU and FNuct and the absence of the FNucs metabolites in the tumours (Figure 7). Spectra *in vitro* demonstrated that FBal was nearly always present following 5FU administration, but overall there was no correlation between FBal concentration and the type of drug regime given to the rat. Table II compares quantitation of the tumour extracts by MRS *in vitro* with the mean peak areas measured *in vivo*. This table

**Figure 2** Peak integrals of 5FU and FNuct in the Walker carcinosarcoma following an i.v. bolus injection of 5FU (50 mg kg$^{-1}$) in the absence and presence of HPP. Rats receiving a molar equivalent dose of HPP were injected i.v. 15 min before 5FU. Results are the mean±s.e. of three tumours (control) and four tumours (HPP). ○, 5FU in controls; ●, FNuct in controls; △, 5FU in HPP-pretreated rats.

**Figure 3** Peak integrals of 5FU and FNuct in the Walker carcinosarcoma following an i.v. bolus injection of 5FU (120 mg kg$^{-1}$) in the absence and presence of HPP. Rats receiving a molar equivalent dose of HPP were injected i.v. 15 min before 5FU. Results are the mean±s.e. of 5 tumours (control) and 4 tumours (HPP). ○, 5FU in controls; ●, FNuct in controls; △, 5FU in HPP-pretreated rats.

**Figure 4** Metabolism by Walker carcinosarcoma of 5FU (120 mg kg$^{-1}$). Rats received an i.v. bolus injection of 5FU at zero time. The receiver gains were already set to maximum and data acquisition began immediately after 5FU injection. The spectral width was 4 kHz and a 1 s repetition time was used. Each spectrum is the sum of four 8 min blocks of 480 transients processed with 19 Hz line broadening. Peaks identified as follows: FU, 5-fluorouracil; FN, 5-fluoronucleotides (deoxy and non-deoxy) and FUDP-sugars.

**Figure 5** Metabolism by Walker carcinosarcoma of 5FU (50 mg kg$^{-1}$) in the presence of HPP. Rats received an i.v. bolus injection of 5FU at zero time, 15 min after a molar equivalent i.v. bolus injection of HPP. The spectral parameters and the processing of results were identical to that described in the legend to **Figure 4**.

**Figure 6** Metabolism by Walker carcinosarcoma of 5FU (120 mg kg$^{-1}$) in the presence of HPP. Rats received an i.v. bolus injection of 5FU at zero time, 15 min after a molar equivalent i.v. bolus injection of HPP. The spectral parameters and the processing of results were identical to that described in the legend to **Figure 4**.

and Figure 8 demonstrate that the areas of the signals (peak integrals) measured by MRS *in vivo* were proportional to the final concentrations measured in the extracts. Thus, increases in the dose of 5FU produced an increase in the FNuct peak integral which reflected significant increases in the FNuct concentrations in the tumour. Similarly, HPP caused a large

**Figure 7** Spectra of extracts from Walker carcinosarcomas freeze-clamped 67 min after a bolus injection of 5FU (120 mg kg$^{-1}$). **a**, 5FU alone; **b**, 5FU 15 min after a molar equivalent dose of 5HPP. The spectral width was 6 kHz. Each spectrum was from 1,500 transients, with an 8.5 s repetition time and was processed with 19 Hz line broadening. Peaks identified as follows: FT, 300 nmol 5-fluorotryptophan; FU, 5-fluorouracil; FN, 5-fluoronucleotides (deoxy and non-deoxy) and FUDP-sugars, FBAL, α-fluoro-β-alanine.

**Figure 8** Peak integrals *in vivo*, *vs*. concentrations of 5FU and FNuct measured in extracts from the Walker carcinosarcoma. Data were used from both the *in vivo* (peak integral) and *in vitro* (quantified concentration) values that applied to each individual tumour. Results are from tumours that received both 50 and 120 mg kg$^{-1}$ 5FU doses in the presence (tests) and absence (controls) of HPP. △, 5FU (controls); ▽, 5FU (tests); ●, FNuct. Slopes were generated using linear correlation, $r$ (5FU) = 0.96; $r$ (FNuct) = 0.89.

decrease in the average peak integral of FNuct, i.e. an 86% and 63% reduction in FNuct concentrations following 50 mg kg$^{-1}$ and 120 mg kg$^{-1}$ 5FU doses, respectively. *In vivo*, the alteration of 5FU netabolism caused by HPP, which affected both the FNuct and 5FU peaks, was most evident as a highly significant decrease in the FNuct/5FU ratio at both of the 5FU doses (Table II).

The FNuct/5FU ratio *in vitro* was always greater than that measured using peak integrals *in vivo* (Table II), probably because the measurements were effectively taken at different times. The peak integral *in vivo* was determined from a mean time of 50 min and the extracts were made at 67 min, immediately after the MRS accumulation was complete. Figures 2 and 3 show that the FNuct levels at both 5FU doses were still increasing at 50 min and the 5FU levels were decreasing. The effect of using these two different time points would also explain why the two slopes in Figure 8 are significantly different ($P<0.1\%$): a peak area of 2,000 represents 100 nmol g$^{-1}$ for 5FU, but 240 nmol g$^{-1}$ for FNuct. Thus the 5FU measurement *in vivo* (area measured at 33–67 min) would be overestimated and the FNuct

**Table II** Comparison of measurements made by MRS *in vivo* and *in vitro* of the levels of 5FU and FNuct in the Walker carcinosarcoma following treatment with 5FU with or without HPP pretreatment

| | *NMR quantitation (*in vitro) | | | | *Peak integrals* (in vivo) | | | |
|---|---|---|---|---|---|---|---|---|
| *Treatment* | *5FU* | *FNuct* (*nmol g*$^{-1}$) | *FBal* | *FNuct/FU ratio* | *5FU* (*arbitrary* | *FNuct* *units*) | *FNuct/FU ratio* | (*n*) |
| 5FU (25 mg kg$^{-1}$) | 0 | 63±21$^c$ | 23±15 | – | 278± 194 | 296±250$^c$ | 1.0±0.9 | (4) |
| 5FU (50 mg kg$^{-1}$) | 24±24 | 172±35 | 22±12 | 7.2 | 746± 431 | 1,478±191 | 1.8±0.4 | (3) |
| 5FU+HPP (50 mg kg$^{-1}$) | 74±28 | 24±24$^a$ | 39±30 | 0.3±0.3 | 1,589± 706 | 34± 34$^a$ | 0.1±0.1$^b$ | (4) |
| 5FU (120 mg kg$^{-1}$) | 202±32 | 273±28 | 78±19 | 1.4±0.2 | 3,945± 595 | 2,328±576 | 0.6±0.1 | (5) |
| 5FU+HPP (120 mg kg$^{-1}$) | 494±26 | 101±39$^b$ | 25± 4 | 0.2±0.1 | 8,112±1,321 | 645±450 | 0.1±0.1$^b$ | (4) |

Samples are the same as in **Figures 2** and 3. Results are mean±s.e. where (*n*) is the number of tumours. The times of the spectra were as follows: *in vivo*, 50 min (33–67 min); *in vitro*, 67 min.
$^a P<0.02$; $^b P<0.01$, comparing test (5FU+HPP) with control (5FU alone); $^c P<0.05$, comparing the low dose of 5FU with the 50 mg kg$^{-1}$ 5FU dose. All comparisons were made using Student's *t* test.

measurement *in vivo* would be underestimated. This artifact, caused by using such a long accumulation time for the MRS data, would cause the regression lines to diverge. Nevertheless, the data in Figure 8 demonstrate that these peak integral values in all tumours for both 5FU and FNuct are directly correlated to the tumour concentrations measured in extracts by MRS *in vitro* ($r=0.96$, 5FU; $r=0.89$, FNuct) and thus increases in peak integrals in the Walker carcinosarcoma reflected increasing intracellular concentrations of 5FU and metabolites. With the shorter accumulation times available at higher fields similar graphs would act as calibration curves.

### *Composition of the FNuct peak*

The hplc-determined FNuct peak presented in Table III consists of the sum of measurements of the tissue concentrations for FUMP, FUDP, and FUTP. The FNuct peak, which has a chemical shift of 4.5–5 p.p.m. *in vivo* (Keniry *et al.*, 1986), may also have contained contributions from FdUMP and FUDP-glucose, but these could not be measured by the method of Prior (1989); this would explain the systematic difference between the hplc-determined FNuct peak and the MRS-determined FNuct peak shown in Table II. Table III demonstrates that FUTP accounts for about 50% of the FNuct signal, which is in good agreement with measurements made in L1210 cells and HCT-8 cells (Cadman *et al.*, 1981; Benz & Cadman, 1981). The proportion is lowered to 13% in tumours from rats receiving HPP 15 min before 5FU.

These data show that lowering the 5FU dose resulted in significantly lower intracellular concentrations of FUTP, but that the drug combination with HPP caused a much larger reduction in FUTP of 94% and 91% for 50 and 120 mg kg$^{-1}$ respectively. There were fairly small differences, however, in the levels of FUMP following treatment with 5FU and HPP compared with 5FU alone (Table III). This is surprising since the locus of HPP action is indirect inhibition of formation of FUMP from 5FU leading to reduced concentrations for FUDP and FUTP (Schwartz & Handschumacher, 1979). It suggests that the conversion of FUMP to FUDP by nucleoside monophosphate kinase has become rate-limiting, perhaps because of competition from other monophosphates, such as orotidine monophosphate, that are increased following HPP metabolism.

### Discussion

5FU is activated by intracellular enzymes to 5-fluoronucleotides (FNuct), two of which (FUTP and FdUMP) are cytotoxic (Heidelberger *et al.*, 1983). In principle MRS could be used to observe FNuct formation non-invasively and thus could be a potential indicator of tumour responsiveness. In practice, however, FdUMP or the ternary complex of FdUMP, thymidylate synthase and the co-factor 5,10,methylenetetrahydrofolate are formed at cytotoxic concentrations that are below the sensitivity of MRS. The ternary complex has been reported to be NMR-visible in

**Table III** Effect of 5FU alone or in combination with HPP on levels of FUTP and FNuct in the Walker carcinosarcoma

| | *Hplc quantitation* | | | | | |
|---|---|---|---|---|---|---|
| *Treatment* | *FUMP* | *FUDP* (*nmol g*$^{-1}$) | *FUTP* | *FNuct* | *FUTP % of NMR-determined FNuct* | (*n*) |
| 5FU (25 mg kg$^{-1}$) | 0 | 7± 4 | 33±12$^c$ | 41±13$^d$ | 54 | (4) |
| 5FU (50 mg kg$^{-1}$) | 18± 3 | 41±12 | 91±11 | 150± 4 | 53 | (3) |
| 5FU+HPP (50 mg kg$^{-1}$) | 16± 9 | 1± 1 | 3± 3$^b$ | 19±11$^b$ | 13 | (4) |
| 5FU (120 mg kg$^{-1}$) | 67±23 | 67±14 | 134±27 | 267±32 | 49 | (5) |
| 5FU+HPP (120 mg kg$^{-1}$) | 43±23 | 24± 8 | 12± 8$^a$ | 78±14$^b$ | 12 | (4) |

Samples are the same as in Table II, i.e. values are determined from tumours freeze-clamped 67 min after treatment with 5FU. Results are mean±s.e. where *n* is the number of tumours.
$^a P<0.02$; $^b P<0.01$ when comparing test (5FU+HPP) with control (5FU alone); $^c P<0.05$; $^d P<0.01$ when comparing the low dose of 5FU with the 50 mg kg$^{-1}$ 5FU dose. All comparisons were made using Student's *t* test.

human pancreatic adenocarcinoma cells but this result was not reproducible and required high doses of 5-fluoro-2′-deoxyuridine *in vitro* (Malet-Martino *et al.*, 1986). Intracellularly, FdUMP can reach concentrations up to 5 nmol g$^{-1}$, which would be sufficient to inhibit thymidylate synthase completely (Berne *et al.*, 1987; Washtein, 1984; Klubes *et al.*, 1978), but which is still below MR sensitivity *in vivo*. In any case, the levels of free intracellular FdUMP may not correlate with cytotoxicity. For example, the Walker carcinosarcoma formed 0.4 nmol g$^{-1}$ FdUMP, four times the concentration synthesised by the 5FU sensitive L1210 cells, but remained relatively insensitive to a single dose of 5FU since it cleared FdUMP very rapidly (Klubes *et al.*, 1978). Similarly, HPP caused a 60% reduction of FdUMP levels in rat colon carcinoma, but did not affect the amount of thymidylate synthase inhibition (Berne *et al.*, 1987). Also, despite thymidylate synthase inhibition, DNA synthesis can continue through salvage of extracellular thymidine (Heidelberger *et al.*, 1983).

The mechanism of 5FU cytotoxicity via FUTP remains less clear. FUTP becomes incorporated into RNA, interfering with RNA methylation, tRNA formation and protein synthesis (Heidelberger *et al.*, 1983). Additionally, F-RNA may persist *in vivo* and lead to a slow release of 5FU over many hours (Spears *et al.*, 1984). Increases in the intracellular concentration of FUTP are associated with increased cell killing in a number of cell lines (Schwartz & Handschumacher, 1979; Cadman *et al.*, 1981; Benz & Cadman, 1981), and FUTP may reach concentrations up to 50 times those of FdUMP (Cadman *et al.*, 1981). These observations suggest not only that FUTP alone would be MRS-visible, but also that changes in its concentration in tumours could be used to predict cytotoxicity.

We have shown using MRS *in vivo* that FNuct was rapidly formed in the Walker carcinosarcoma following a single 5FU dose at and above 50 mg kg$^{-1}$. Analysis of the extracts from these tumours by MRS *in vitro* and by hplc demonstrated that lowering the 5FU dose to 25 mg kg$^{-1}$, or using combination chemotherapy with HPP, decreased the intracellular concentration of FNuct. Hplc showed that 50% of this FNuct in controls was the cytotoxic species FUTP. Compared to the 50 mg kg$^{-1}$ dose of 5FU, the low dose of 5FU produced 64% less intracellular FUTP, while co-administration of HPP caused a 90% reduction in FUTP levels. These large changes in FNuct which can be measured quantitatively *in vitro* at a single time-point (in this case 67 min) could also be detected *in vivo*, as a highly significant change in the FNuct/5FU peak integral ratio. Furthermore, under the conditions used in these experiments, the levels of FNuct and 5FU formed *in vivo* could be estimated since the peak integrals were shown to be proportional to the final concentrations measured in the extracts at 67 min. Drug regimes that produced a mean FNuct peak integral below 300 arbitrary units (from Figure 8 this would be $<35$ nmol g$^{-1}$) were associated with reduced cytotoxicity since they did not cause significant inhibition of tumour growth. Included in these drug regimes was the 5FU (50 mg kg$^{-1}$) + HPP combination which produced less FUTP than the 5FU (25 mg kg$^{-1}$) alone (Table III), even though the former regime seemed more cytotoxic (Table I). Since the locus of HPP action is to inhibit the formation of FUMP this might encourage increased metabolism of 5FU towards FdUMP if the levels of deoxyribose-1-phosphate are sufficiently high (Figure 1), leading to a higher proportion of DNA-directed cytotoxicity in the presence of HPP. However, as discussed above, this mode of cytotoxicity may not be significant in the Walker carcinosarcoma (Klubes *et al.*, 1978), and it could be that at these lower concentrations FUTP is rapidly incorporated into RNA where it is undetectable by our methods.

Using MRS *in vivo*, different levels of FNuct formation have been reported in murine tumours that have differential sensitivity to 5FU (Hull *et al.*, 1986). In this case FNuct formation was observed in the '5FU-resistant' M5076 cells but was absent in the '5FU-sensitive' sarcoma 180 cells. However, in this experiment the S180 tumours were highly necrotic and the M5076 cells were mostly viable and later results with viable tumours suggested a higher anabolite/catabolite ratio for S180 compared to M5076 (W. Hull, personal communication).

5FU was immediately and clearly visible in tumours at both of the high doses of 5FU. This signal declined at a faster rate than the FNuct signal increased probably because 5FU underwent catabolism and efflux from the tissue as well as anabolism. However, in the presence of HPP the disappearance of 5FU from tumours was slower. HPP may reduce hepatic clearance of 5FU in patients (Howell *et al.*, 1981) and these results suggest an additional effect on tumour catabolism. Signals corresponding to FNucs were never observed by MRS *in vivo* or *in vitro* (detection limit *in vitro* was 10 nmol g$^{-1}$) and could not be distinguished from other nucleotides on our ion-exchange hplc. Of the possible catabolic products from 5FU metabolism only FBal was seen *in vivo*, but not consistently. Analysis of extracts showed that in fact FBal was present at all doses of 5FU at concentrations ranging from a mean of 20 up to 80 nmol g$^{-1}$, suggesting that MRS resolution *in vivo* was limited by the broadness of the signal and/or the effect of saturation due to a long $T_1$ for FBal *in vivo*. Yet in the extracts there was no correlation between FBal concentrations and the concentrations of FNuct or 5FU. It is possible the FBal we observe owes its source to the rapid 5FU catabolism that occurs in rat liver (Cabanac *et al.*, 1988) and it may be extracellular. So, neither FBal peaks *in vivo*, nor FBal concentrations *in vitro*, are related to 5FU cytotoxicity. These observations are similar to that made by Hull *et al.* (1988), who did not find a correlation between clinical success and the pharmacokinetics of 5FU catabolism in patients receiving 5FU or 5FU plus methotrexate chemotherapy.

The use of peak integrals to estimate the concentration of drugs *in vivo* may only be appropriate in our experimental protocol and probably could not universally be applied. It does suggest, however, that at higher fields and with the development of quantitative methods, MRS could be used as a predictive tool by the clinician. It is also pertinent to note that we have not determined whether multiple treatments of the same animal would result in the 5FU metabolic profiles we have described here. Nevertheless, our study of the MRS signals observed in the Walker carcinosarcoma following an injection of 5FU or HPP and 5FU, shows that only the FNuct peak integral predicted which drug regime was likely to induce tumour regression. To our knowledge this is the first demonstration that the size of the FNuct peak is relevant to determining the cytotoxicity of 5FU towards tumours. Our results suggest that MRS *in vivo* could assess the sensitivity of tumours that are responsive to 5FU *via* the cytotoxic species FUTP. Furthermore, it has been shown that the mechanism of action of 5FU can be directed towards FUTP and RNA cytotoxicity by using methotrexate (Benz & Cadman, 1981) or thymidine (Takimato *et al.*, 1987). It is therefore possible that MRS could be used to determine the optimal scheduling of this type of combination chemotherapy. These possibilities are currently under investigation.

This work was supported by the Cancer Research Campaign.

### References

BENZ, C. & CADMAN, E. (1981). Modulation of 5-fluorouracil metabolism and cytotoxicity by antimetabolite pretreatment in human colorectal adenocarcinoma HCT-8. *Cancer Res.*, **41**, 994.

BERNE, M., GUSTAVSSON, B., ALMERSJO, O., SPEARS, C.P. & WALDENSTROM, J. (1987). Concurrent allopurinol and 5-fluorouracil: 5-fluoro-2′-deoxyuridylate formation and thymidylate synthase inhibition in rat colon carcinoma and in regenerating rat liver *Cancer Chemother. Pharmacol.*, **20**, 193.

CABANAC, S., MALET-MARTINO, M.C., BON, M., MARTINO, R., NEDELEC, J.F. & DIMICOLI, J.L. (1988). Direct $^{19}$F-NMR spectroscopic observation of 5-fluorouracil metabolism in the isolated perfused mouse liver model. *NMR Biomed.*, **1**, 113.

CADMAN, E., HEIMER, R., & BENZ, C. (1981). The influence of methotrexate pre-treatment of 5-fluorouracil metabolism in L1210 cells. *J. Biol. Chem.*, **256**, 1695.

GRIFFITHS, J.R., BHUJWALLA, Z., COOMBES, R.C. and 10 others (1987). Monitoring cancer therapy by NMR spectroscopy. *Ann. NY Acad. Sci.*, **508**, 183.

HEIDELBERGER, C., DANENBERG, P.V. & MORAN, R.G. (1983). Fluorinated pyrimidines and their nucleosides. *Adv. Enzymol.*, **54**, 57.

HOWELL, S.B., WALLACE, M.D., WUNG, E., TAETLE, R., HUSSAIN, F. & ROMINE, J.S. (1981). Modulation of 5-fluorouracil toxicity by allopurinol in man. *Cancer*, **48**, 1281.

HULL, W.E., PORT, R.E., OSSWALD, H. & KUNZ, W. (1986). *In vivo* $^{19}$F-NMR study of 5-fluorouracil metabolism in liver and implanted tumours of the mouse. *Abstracts of the 5th Annual Meeting, Soc. Magn. Res. Med., Montreal*, p. 594.

HULL, W.E., PORT, R.E., KUNZ, W. & SCHLAG, P. (1987). $^{19}$F-NMR for monitoring 5-fluorouracil chemotherapy. *J. Cancer Res. Clin. Oncol.*, **113**, S46.

HULL, W.E., PORT, R.E., HERRMANN, R., BRITSCH, B. & KUNZ, W. (1988). Metabolites of 5-fluorouracil in plasma and urine as monitored by $^{19}$F-NMR, for patients receiving chemotherapy with or without methotrexate pretreatment. *Cancer Res.*, **48**, 1680.

KENDALL, M.G. & STUART, A. (1968). *The Advanced Theory of Statistics*, vol. 3, 2nd ed, p. 45.

KENIRY, H., BENZ, C., SHAFER, R.H. & JAMES, T.L. (1986). Non-invasive spectroscopic analysis of fluoropyrimidine metabolism in cultured tumour cells. *Cancer Res.*, **46**, 1745.

KLUBES, P., CONNELLY, K., CERRA, I. & MANDEL, H.G. (1978). Effects of 5-fluorouracil on 5-fluorodeoxyuridine 5′-monophosphate and 2-deoxyuridine 5′monophosphate, and DNA synthesis in solid mouse L1210 and rat Walker 256 tumours. *Cancer Res.*, **38**, 2325.

KOUTCHER, J.A., BARNETT, D.C., MARTIN, D.S., STOLFI, R., SAWYER, R. & COWBURN, D. (1987). *In vivo* $^{19}$F-NMR studies of agents altering 5-fluorouracil metabolism. *Abstracts of the 6th Annual Meeting, Soc. Magn. Res. Med., New York*, p. 108.

MACKINTOSH, J. & TATTERSALL, M.H.N. (1987). Biochemical modulation of 5-fluorouracil therapy in advanced colorectal cancer. *Ann. Acad. Med.*, **16**, 444.

MALET-MARTINO, M.-C., FAURE, F., VIALANEIX, J.-P., PALEVODY, C., HOLLANDE, E. & MARTINO, R. (1986). Non-invasive fluorine-19 NMR study of fluoropyrimidine metabolism in cell cultures of human pancreatic and colon adenocarcinoma. *Cancer Chemother. Pharm.*, **18**, 5.

PINEDO, H.M. & PETERS, G.F.J. (1988). Fluorouracil: biochemistry and pharmacology. *J. Clin. Oncol.*, **6**, 1653.

POGOLOTTI, A.L., NOLAN, P.A. & SANTI, D.V. (1981). Methods for the complete analysis of 5-fluorouracil metabolites in cell extracts. *Anal. Biochem.*, **117**, 178.

PRIOR, M.J.W., McSHEEHY, P.M.J., MAXWELL, R.J. & GRIFFITHS, J.R. (1987). $^{19}$F-NMR studies of the effect of allopurinol on 5-fluorouracil metabolism *in vivo* and *in vitro*. *Abstracts of the 6th Annual Meeting, Soc. Magn. Res. Med., New York*, p. 502.

SCHWARTZ, P.M. & HANDSCHUMACHER, R.E. (1979). Selective antagonism of 5-fluorouracil cytotoxicity by allopurinol *in vitro*. *Cancer Res.*, **39**, 3095.

SEMMLER, W., BACHERT-BAUMANN, P., GUCKEL, F., LEHNER, B., SCHLAG, P. & V. KAICK, G. (1988). Non-invasive monitoring of 5-fluorouracil catabolism and anabolism in patients by means of $^{19}$-MRS: comparison of intraarterial and intravenous administration. *Abstracts of the 7th Annual Meeting, Soc. Magn. Res. Med. San Francisco*, p.258.

SPEARS, C.P., SHANI, J., SHAHINIAN, A.H., WOLF, W., HEIDELBERGER, C. & DANENBERG, P.V. (1984). Assay and time-course of 5-fluorouracil incorporation into RNA of L1210/0 ascites cells *in vivo*. *Molec. Pharmacol.*, **27**, 302.

STEVENS, A.N., MORRIS, P.G., ILES, R.A., SHELDON, P.W. & GRIFFITHS, J.R. (1984). 5-Fluorouracil metabolism monitored *in vivo* by $^{19}$F-NMR. *Br. J. Cancer*, **50**, 113.

STUBBS, M., RODRIGUES, L.M. & GRIFFITHS, J.R. (1988). Correlation of $^{31}$P-NMR spectra with acid extracts and histology in a growth study of some animal tumours. *Abstracts of the 7th Annual Meeting, Soc. Magn. Res. Med., San Francisco*, p. 407.

TAKIMOTO, C.H., TAN, Y.Y., CADMAN, E.C. & ARMSTRONG, R.D. (1987). Correlation between ribosomal RNA production and RNA-directed fluoropyrimidine cytotoxicity. *Biochem. Pharamacol.*, **36**, 3243.

WASHTEIN, W.L. (1984). Comparison of 5-fluorouracil metabolism in two gastrointestinal tumour cell lines. *Cancer Res.*, **44**, 909.

WOLF, W., ALBRIGHT, M.J., SILVER, M.S., WEBER, H., REICHARDT, U. & SAUER, R. (1987). Fluorine-19 NMR spectroscopic studies of the metabolism of 5-fluorouracil in the liver of patients undergoing chemotherapy. *Magn. Reson. Imaging*, **5**, 165.